# The Predictive Value of Head Circumference Growth during the First Year of Life on Early Child Traits

**DOI:** 10.1038/s41598-018-28165-8

**Published:** 2018-06-29

**Authors:** Caroline Dupont, Natalie Castellanos-Ryan, Jean R. Séguin, Gina Muckle, Marie-Noëlle Simard, Gabriel D. Shapiro, Catherine M. Herba, William D. Fraser, Sarah Lippé

**Affiliations:** 1 University of Montreal, Montreal, Canada; 2 Research Center of the Sainte-Justine University Hospital, Montreal, Canada; 3 Université Laval, Quebec, Canada; 4 Quebec CHU-Laval University Research Center, Quebec, Canada; 5 McGill University, Montreal, Canada; 6 Université du Québec à Montréal (UQÀM), Montreal, Canada; 7 Université de Sherbrooke, Sherbrooke, Canada; 8 Research Center of Sherbrooke University Hospital, Sherbrooke, Canada

## Abstract

Atypical head circumference (HC) growth has been associated with neurodevelopmental disorders. However, whether it is associated with specific aspects of development in early childhood in the general population is unknown. The objective of this study was to assess the predictive value of HC growth as an early biomarker of behavioral traits. We examined longitudinal associations between HC growth from 0 to 12 months and temperament, cognitive, and motor development at 24 months. A subsample of healthy children (N = 756) was drawn from the 3D (Design, Develop, Discover) cohort study. Early HC growth was modeled with latent growth curve analysis. Greater postnatal HC growth predicted lower temperamental effortful control and lower surgency/extraversion in boys. HC growth did not predict cognitive or fine motor scores, but did predict greater gross motor skills in boys. No significant effect of HC growth was found in girls. This study is the first to demonstrate an association between postnatal HC growth and specific aspects of child development in a healthy population. Results suggest HC growth overshadows brain mechanisms involved in behavioral traits in early infancy. Whether links are maintained throughout development and the mechanisms involved correspond to traits found in atypical populations remains to be studied.

## Introduction

Anomalies in structural and functional development of the brain have been associated with characteristics of certain neurodevelopmental disorders and impairments in child development^1–4^. Genetic and biological mechanisms determine prenatal and postnatal brain growth^5^. Genetic profile can alter important biological mechanisms involved in brain growth, resulting in microcephaly or macrocephaly. A number of genetic syndromes involving learning disabilities, autistic and attention deficit or hyperactivity traits are associated with abnormal brain growth. Patients with Angelman and Rett syndromes typically exhibit microcephaly^6,7^, while Phosphatase and tensin homolog (PTEN) -related disorders and Tuberous Sclerosis Complex patients tend to show macrocephaly^8,9^. Some evidences point toward abnormal cerebral growth patterns in autism spectrum disorder (ASD), attention deficit/hyperactivity disorder (ADHD) and motor delays. However, studies pertaining to brain volume and growth have yielded inconsistent results^10–16^. In particular, the direction of the link is a matter of debate. For example, reported genes represented in ASD populations were shown to cause increased or decreased brain size^17^. Nevertheless, head circumference (HC) is in general significantly larger in autistic compared to neurotypical individuals, and even more so during early childhood^18^. Retrospective studies of infants who later developed attention deficit/hyperactivity disorder (ADHD) have shown slower increase in HC^16^ and smaller HC persisting as far as 18 months of age^14^. Of note, Raghuram, Yang^15^ also found that in infants born preterm, lower HC growth was associated with greater neurodevelopmental impairments, specifically cognitive and motor delays. Thus, abnormally slow or fast head size growth has been highlighted in neurodevelopmental disorders involving socio-emotional, cognitive and motor impairments. However, whether brain growth relates to specific behavioral traits pertaining to children’s socio-emotional, cognitive and/or motor development in the general population is unknown.

Domains examined to measure early childhood development and outline behavioral traits most commonly include socio-emotional, cognitive, and motor development. Although socio-emotional adjustment in early childhood can be conceptualized in several ways, studies have often focused on the assessment of temperament. Temperament core dimensions includes negative affect, surgency/extraversion and effortful control which, respectively, have been shown to confer risk for behavioral problems and affective disorders^19–21^, communication development and children’s conversational skills^22,23^, and increased risk for the development of externalizing disorders and problems related to attention^19,24,25^. Finally, the literature on early childhood development has also consistently identified poor cognitive and motor abilities in young children as a shared risk factor in a plethora of neurodevelopmental disorders^26–28^. Research shows that better cognitive abilities are related to higher social and academic competence in young children^29,30^ and lower risk for internalizing and externalizing behavioral problems^31^. Early motor abilities are significantly related to language development^32^, social skills^33^ and executive functions development in later childhood^34^. Hence, temperament, cognitive and motor abilities are tracked as indicators of early childhood development. While brain size is still under study to verify whether it is a relevant biomarker of neurodevelopment in clinical populations, the study of healthy general population brain size variations could further be indicative of the directions of associations between brain size and specific behavioral traits in children.

Brain volume more than doubles during the first year of life and reaches almost adult size by 2 years of age^18–20^. A wide range of methods are available to assess brain growth during infancy, but clinicians and researchers agree that head circumference (HC) measurement is the most simple, rapid and inexpensive tool to assess brain size and to identify neonates with abnormal brain growth patterns^18,19^. The particularly intense brain growth happening during the first postnatal months is paralleled by an equally intense increase of HC^35^. Although HC measures skull size, genetic studies have shown it is highly correlated with intracranial volume, which closely reflects brain volume during infancy^36^. Hence, more knowledge regarding if and how deviations in HC growth trajectory relate to early childhood development and specific behavioral traits could improve pediatric health care.

The objective of this study was thus to investigate the predictive value of HC growth trajectory in the first year of life on early child development at 24 months of age in a healthy general population sample. More specifically, temperament, cognitive skills and motor skills at 24 months were assessed. Based on the literature reviewed, it was hypothesized that there would be an association between atypical HC growth (either more rapid or slower) and child development at 24 months.

## Method

### Participants

#### 3D Cohort

**Figure 1:**
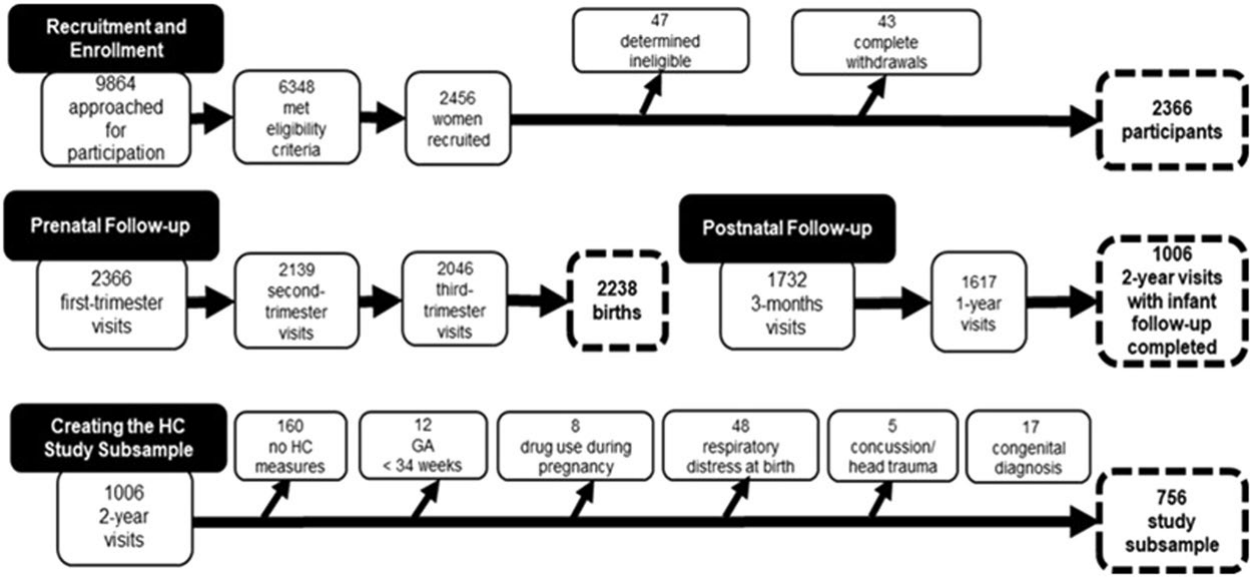
Recruitment and Follow-up in the 3D Study and Creating the Head Circumference Subsample. Note: GA = Gestational age.

A subsample of participants (N = 756) were drawn from the large-scale longitudinal Design, Develop, Discover (3D) cohort study^37^, which includes prospectively-collected data, and aims to address prenatal and early-life determinants of perinatal health and child development. Participants were recruited at nine urban clinical centers in three metropolitan areas in the province of Quebec (Canada), accounting for more than half of the population of Quebec. 2366 pregnant women, between 18 and 45 years of age, participated in the study (Fig. 1). They were recruited during the first trimester of pregnancy (6 to 14 gestational weeks), seen during the second (20 to 24 gestational weeks) and third trimester (32 to 35 gestational weeks), and followed along with their child at birth and at 3, 12 and 24 months postpartum (see^37^ for more details on the recruitment process). The study was approved by the CHU Sainte-Justine Research Ethics Board and by the ethics board of each participating study center. All experiments were performed in accordance with relevant guidelines and regulations. Informed consent was obtained from all participants.

#### Study Subsample

A subsample was selected from the 3D cohort study (Fig. 1) to conduct analyses. Participants for whom data on child behavior, temperament, cognitive skills and motor skills were not collected at the 24-month infant follow-up visit (n = 1360) and those for whom no HC measures were available at any time point were excluded (n = 160). Since the aim of the study was to investigate HC growth and general child development in a non-clinical population, infants with serious health conditions, neurological insults or known risk factors that could affect normal development were excluded from the study subsample. Thus, infants whose mother used drugs during pregnancy (n = 8) and infants born before 34 weeks of gestational age (n = 12), who suffered from respiratory distress at birth (n = 48), who had a history of concussion and head trauma (n = 5) or a diagnosis of congenital abnormalities (n = 17) were excluded. This resulted in a final study subsample size of 756 that was subsequently divided in two subsamples based on infant sex (male, n = 375; female n = 381) for analyses, as norms for HC and HC growth show differentiated development for girls and boys^35^.

### Measures

Mothers completed questionnaires prenatally (at each trimester) and postnatally regarding socio-demographic information and medical history of their child. Follow-up visits with infants were performed at 3, 12, and 24 months of age. All measures used in the current study were drawn from the questionnaires completed prenatally and postnatally by the mother regarding sociodemographic information, medical history, child temperament, and from the evaluation of the infant’s cognitive and motor abilities conducted by a qualified health professional at 24 months postpartum.

#### HC Measurements

Information regarding HC measurements were drawn from the medical history questionnaires completed at birth, 3 months and 12 months postpartum. These measurements were used to model HC growth trajectories.

#### Temperament

Child temperament was assessed at 24 months using the Very Short Form of the Early Childhood Behavior Questionnaire (ECBQ^38^). The ECBQ is a well-established, reliable, and valid measure of child temperament^39^. The very short form, comprising 36 items rated on a scale of 1 (extremely untrue of your child) to 7 (extremely true of your child), has been proven useful for screening and detecting the more salient aspects of temperament and those related to psychopathology. This tool allows for characterization of early child temperament using three broad subscales: Surgency/Extraversion (SE), which refers to the tendency to act with impulsive and active behavior including positive affect; Effortful Control (EC) which involves self-regulation, including voluntary regulation of attention and behavior; and Negative Affect (NA) which is characterized by the predisposition to experience negative feelings and difficulty being soothed^40^. These three broad temperamental traits have consistently been identified in factor analyses of early childhood temperament^40–42^.

#### Cognitive and Motor Development

Infant cognitive and motor development were evaluated using the Bayley Scales of Infant and Toddler Development, Third Edition (Bayley-III^43^). The Bayley-III is designed to measure the developmental functioning of infants and toddlers and to identify possible developmental delay. The Cognitive scale of the Bayley-III contains 91 items assessing information processing, conceptual resources and perceptual skills. The Motor scale of the Bayley-III consists of Fine Motor and Gross Motor subtests. The Fine Motor subtest contains 66 items measuring skills associated with eye movements, perceptual-motor integration, motor planning, and motor speed. The Gross Motor subtest contains 72 items assessing movements of the limbs and torso. One parent, usually the mother, completed the Modified Checklist for Autism in Toddlers (M-CHAT^44^); a screening tool designed to identify children showing possible early signs of ASD or developmental delay. In the present study, it is used to complement the assessment of child development.

#### Covariates

Infant gestational age at birth (GA), family income (dichotomous variable based on Statistics Canada’s low-income cut-off for family size) and maternal education (number of completed years of schooling) were used as covariates in all analyses. Family income and maternal education are commonly used indicators of socioeconomic status (SES). GA has been shown to influence HC at birth^45^ and SES is a factor known for influencing both general child development and brain development^46,47^.

### Statistical Analyses

#### Preliminary Analyses and Descriptive Statistics

Analyses were carried out using IBM SPSS for Windows, Version 22.0. The amount of partial missing data ranged from 3% to 41% (Bayley-III cognitive and motor development at 24 months follow-up). When separating the sample by sex, the amount of missing data was similar for both boys and girls. Descriptive analyses were performed separately for boys (n = 375) and girls (n = 381) subsamples.

#### Unconditional Latent Growth Curve Models

Analyses were carried out with latent growth curve and path analyses using Mplus version 7 for Windows. Maximum likelihood with robust standard errors (MLR) estimation was used in all analyses and full information maximum likelihood (FIML) was used to account for missing data. First, several unconditional latent-growth curve models (LGCM) were conducted to assess HC development during the first year of life, modeling both the intercept (HC at birth) and slope (HC growth from 0 to 12 months). LGCM methodology is substantially more flexible than traditional analysis of variance for the assessment of longitudinal data, as it allows examining intra-individual change and inter-individual differences in change across time^48^ using all data available.

**Figure 2:**
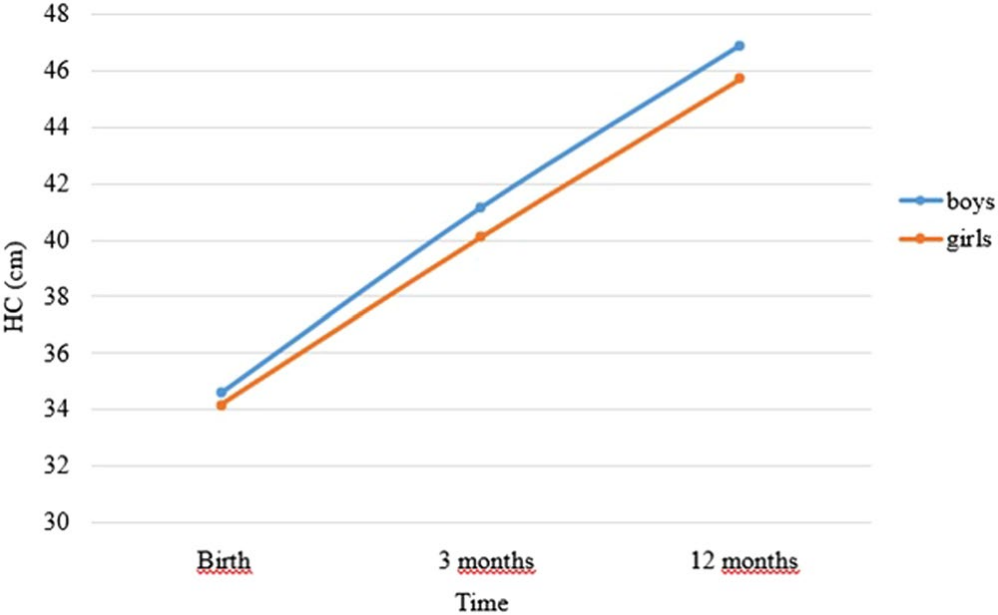
Growth in Head Circumference (HC) from Birth to 12 months.

All models were run separately for girls (n = 381) and boys (n = 375), as multigroup models, in Mplus. A model assuming linear growth in HC across the first year of life (time score loadings constrained at 0, 1 and 2) for both boys and girls did not fit the data well (X2 (2, 756) = 25.43 (boys = 20.45; girls = 4.98), CFI = 0.88, TLI = 0.63, RMSEA = 0.176, SRMR = 0.18). Thus, a second model was constructed in which the loading for the last time point was freed for boys and girls. In order to identify the model (having at least 1 degree of freedom), the residual variance for the first time-point was constrained to be equal across infant sex. This was deemed justified as the residual variance for the first-time point was comparable for boys (1.30, p < 0.0001) and girls (1.16, p < 0.001). This model fit the data very well (X2 (1, 756) = 0.005 (boys = 0.003; girls = 0.002), CFI = 1.00, TLI = 1.03, RMSEA = 0.00, SRMR = 0.01), and showed that the mean HC at birth was slightly larger for boys (34.57 cm, p < 0.001) than for girls (34.16 cm, p < 0.001) and average overall growth in HC from birth to 12 months was larger in boys (slope mean = 6.55, p < 0.001) than girls (slope mean = 5.92, p < 0.001). The freely estimated loading for the last time point indicated that growth slowed down slightly more for boys (time score loading at 24 months (last time point) of 1.88) than for girls (time score loading at 24 months of 1.95, which approximates a loading indicating linear growth, i.e., a loading of two) from 3 to 12 months of age (see Fig. 2). Results also showed that the variance for the intercept and slope factors of HC was significant in both boys (intercept: 1.06, p < 0.001; slope: 0.33, p = 0.001) and girls (intercept: 0.96, p < 0.001; slope: 0.19, p = 0.017), indicating that there was significant individual variability in HC at birth and in HC growth over the first 12 months of life in this sample. Finally, HC at birth was negatively associated with growth in HC from birth to 12 months (boys: r = −0.31, p = 0.052; girls: r = −0.32, p = 0.011).

#### Path Models Testing Associations between HC Factors and Child development Outcomes at 24 months

Once head circumference development was modeled, the LGCM factors for HC (HC at birth and HC growth during the first year of life) together with covariates (GA, family income and maternal education) were included into one multivariate path model to test the relationship between HC development and child development outcomes at 24 months (temperament, cognitive and motor).

### Data availability

The data that support the findings of this study are available from IRNPQEO but restrictions apply to the availability of these data, which were used under license for the current study, and so are not publicly available. Data are however available from the authors upon reasonable request and with permission of IRNPQEO.

## Results

### Descriptive Statistics and Preliminary Analyses

Table 1 presents the means and standard deviations of each continuous variable used to describe the boys and girls subsamples. In the sample, 49% of infants were male. The mean GA at birth was 39.0 weeks (SD = 1.4) for boys and 39.2 weeks (SD = 1.3) for girls. The mean maternal education level were 17.3 years (SD = 3.0) and 16.9 years (SD = 2.7), for the boy and girl subsamples respectively. Furthermore, 9.3% of participants in the boys subsample and 9.4% in the girls subsample had a before-tax household income below Statistics Canada’s before-tax low-income cut-off for their family size, which is a little lower than the 2010 Canadian norm of 13.7%. The mean HC at all 3 time points (birth, 3 months, 12 months) was similar to established norms^49^. Child development outcomes (ECBQ, Bayley and M-CHAT scores) tended to cluster around normal values in both sex subsamples. Screening of variable distributions revealed normal or near-normal distributions (all kurtosis and skewness indices ≤2.5).

**Table 1:** Means and Standard Deviations (SD) of Head Circumference (HC) and Developmental Outcomes.

	Boys (N = 375)		Girls (N = 381)	
	n	Mean (SD)	n	Mean (SD)
**HC (cm)**				
Birth	363	34.6 (1.5)	371	34.2 (1.4)
3 months	338	41.1 (1.4)	355	40.1 (1.2)
12 months	335	46.9 (1.3)	358	45.6 (1.2)
**Developmental outcomes at 24 months**				
Surgency/Extraversion	330	4.0 (1.0)	327	3.6 (1.0)
Negative Affect	330	2.2 (0.6)	327	2.3 (0.7)
Effortful control	330	4.9 (0.7)	327	5.0 (0.7)
Cognitive skills	237	9.9 (2.0)	235	10.0 (2.1)
Fine motor skills	223	11.4 (2.6)	222	11.7 (2.9)
Gross motor skills	223	8.8 (2.1)	222	9.3 (2.6)
M-CHAT	330	0.8 (1.0)	327	0.8 (1.0)

### Path Models Testing Associations between HC Factors and Child Development Outcomes at 24 months

The univariate latent growth model for HC was included in a model with child development outcomes at 24 months (temperament, cognitive development, fine and gross motor development and M-Chat) to test the relationship between both HC at birth (intercept) and HC growth during the first year of life (slope), and child development, controlling for gestational age and SES (family income and maternal education). Thus, regression paths from the intercept and slope latent factors for HC to these outcome variables were included in this model. Table 2 shows regression coefficients from HC at birth and HC growth during the first year of life to child development outcomes at 24 months. Results indicate that, controlling for covariates, HC growth during the first year of life significantly predicted the temperamental traits of surgency and effortful control, as well as gross motor skills in boys, but failed to predict cognitive development, fine motor skills and M-CHAT scores. Furthermore, HC growth during the first year of life failed to significantly predict any outcomes in girls. HC at birth did not predict child development outcomes at 24 months, neither in boys, nor in girls. None of the covariates (GA, family income and maternal education) significantly predicted child development outcomes at 24 months.

**Table 2:** Head Circumference and Covariates Predicting Developmental Outcomes at 24 months.

	Surgency		Effortful Control		Negative Affect		Cognitive skills		Gross motor skills		Fine motor skills		M-CHAT	
	B (SE)	β	B (SE)	β	B (SE)	β	B (SE)	β	B (SE)	β	B (SE)	β	B (SE)	β
**Boys**														
HC at birth	0.00 (0.10)	0.00	0.12 (0.09)	0.17	−0.02 (0.05)	−0.02	−0.17 (0.22)	−0.09	−0.26 (0.27)	−0.12	−0.36 (0.31)	−0.14	−0.04 (0.09)	−0.05
HC growth 0–12 months	−0.44 (0.23)	−0.24*	−0.36 (0.18)	−0.23*	0.07 (0.10)	0.06	0.43 (0.44)	0.12	1.15 (0.56)	0.31*	0.69 (0.68)	0.15	0.00 (0.18)	0.00
Gestational age	−0.08 (0.08)	−0.10	−0.14 (0.07)	−0.25	0.01 (0.04)	0.03	0.15 (0.19)	0.10	0.34 (0.25)	0.22	0.44 (0.27)	0.23	−0.01 (0.08)	−0.02
Income status	−0.02 (0.19)	−0.01	−0.25 (0.14)	−0.10	0.05 (0.14)	0.02	0.18 (0.47)	0.03	0.22 (0.49)	0.03	−0.19 (0.58)	−0.02	0.22 (0.18)	0.06
Maternal education	−0.03 (0.05)	−0.03	−0.02 (0.04)	−0.03	−0.04 (0.03)	−0.08	−0.14 (0.11)	−0.07	0.19 (0.15)	0.10	0.02 (0.16)	0.01	−0.05 (0.06)	−0.06
**Girls**														
HC at birth	−0.03 (0.09)	−0.03	0.15 (0.08)	0.20	−0.03 (0.06)	−0.05	0.14 (0.21)	0.06	−0.11 (0.31)	−0.04	−0.37 (0.35)	−0.13	0.03 (0.09)	0.03
HC growth 0–12 months	0.11 (0.26)	0.05	−0.39 (0.29)	−0.24	0.31 (0.22)	0.20	0.14 (0.61)	0.03	1.43 (1.07)	0.24	0.10 (1.04)	0.16	0.16 (0.24)	0.07
Gestational age	0.01 (0.08)	0.01	−0.11 (0.08)	−0.20	0.03 (0.06)	0.07	0.04 (0.23)	0.03	0.39 (0.32)	0.21	0.58 (0.33)	0.28	0.02 (0.07)	0.03
Income status	0.51 (0.18)	0.16	0.13 (0.14)	0.05	0.38 (0.16)	0.16	−0.34 (0.50)	−0.05	1.36 (0.62)	0.15	0.34 (0.61)	0.03	0.42 (0.25)	0.12
Maternal education	0.01 (0.03)	0.02	0.08 (0.04)	0.13	0.02 (0.03)	0.04	0.06 (0.10)	0.03	0.02 (0.15)	0.01	0.20 (0.16)	0.08	0.06 (0.05)	0.07

Note: *p < 0.05, HC = Head Circumference; Model fit for a) the model predicting temperament: X2 (13, 756) = 21.83 (boys = 16.62; girls = 5.20); CFI = 0.99; TLI = 0.92; RMSEA = 0.04; SRMR = 0.06; b) the model predicting Bayley’s and M-CHAT scores: X2 (15, 756) = 20.54 (boys = 15.29; girls = 5.26); CFI = 0.99; TLI = 0.99; RMSEA = 0.03; SRMR = 0.06.

## Discussion

To our knowledge, this is the first study in a general population sample to longitudinally examine associations between head growth during the first year of life and infants’ behavioral traits. Our results demonstrated that postnatal HC growth negatively predicted temperamental surgency/extraversion and effortful control, and positively predicted gross motor skills at 2 years old. This predictive value was exhibited for boys only.

Temperament refers to individual differences in reactivity and regulation that are thought to be neurobiologically based and serve as the foundation for subsequent personality^50,51^. Surgency, which includes high-level of energy and positive approach behaviors, is associated with better communicative skills in toddlers, and with extraversion in childhood^52^. In clinical practice, temperamental profiles including less positive anticipation^53,54^, less enjoyment in play^55^ and less surgency^56^ are found in toddlers later diagnosed with ASD. Our results showed that slower HC growth during the first year of life is related to higher level of surgency in later development, whereas a more rapid increase in HC is a predictor for lower tendency for outgoing behaviors/interactions during early childhood. At first glance, these results thus seem in line with the existing, yet inconsistent, literature on the association between increased brain growth and ASD^10,48–50^. However, in our study, HC growth during the first year of life did not predict M-CHAT scores. Exclusion criteria for our subsamples were most of the contributing factors to neurodevelopmental disorders and, accordingly, most of our participants scored very low on the M-CHAT questionnaire. Thus, our results suggest that rapid HC growth associated with low temperamental surgency domain is not specific to ASD diagnosis, but could be more relevant to general long-term social adaptation. Recent findings suggesting that significant low levels of surgency in ASD toddlers do not predict ASD severity levels^57^ seem to support this view. Hence, brain growth during the first year of life is seemingly a relevant biomarker of surgency in the general population, and remains to be more carefully explored in clinical populations as a specific phenotypic profile within a broad spectrum.

Effortful control, which includes self-regulation of attention and behavior, is negatively associated with several psychopathology and neurodevelopmental disorders involving internalizing and externalizing symptoms^21,58^. Lack of effortful control is associated with later ADHD of both types^59^. Here, slower HC growth was shown to be protective of effortful control temperamental domain. This result is surprising given that retrospective studies of infants who later developed attention deficit/hyperactivity disorder (ADHD) showed slower HC increase^16^ and smaller HC persisting as far as 18 months of age^14^. Yet, lack of effortful control is present in several neurodevelopmental disorders, including ASD^56^. Our results come from a general population where HC growth trajectories followed a normal distribution and known risk factors were excluded. Several factors, including genetic and environmental factors, may account for a relationship between brain size and behavior in the clinical or the general population. From an evolutionary point of view, the grade shifts from monkeys to apes and from apes to humans are associated with differences in neural development and patterns of brain growth^60,61^. Hence, slower cerebral development appears necessary to the emergence of higher-order cognitive skills characterizing humans. Brain regions associated with higher order functions such as attention, executive control and emotion regulation exhibit a protracted developmental timetable^62,63^. For example, effortful control, which includes the ability to inhibit dominant responses to perform subdominant responses^40^, is thought to be closely related to underlying brain networks of executive attention in the anterior cingulate and other frontal areas^63,64^. In an infant brain where structural connections of myelinated white matter are slowly maturing^65,66^, slower brain growth and smaller brain volumes seem beneficial for these higher-order networks, as it might allow their underlying brain structures to more optimally integrate, rather than leaving the networks to segregated regions^67,68^.

Conversely, faster brain growth and bigger brain volumes could be beneficial for brain regions developing early, such as the sensorimotor and visual cortex^69–71^. Our results showed faster HC growth predicted greater gross motor skills, such as motor coordination, motor planning and balance. Networks underlying these functions are the fastest to myelinate, facilitating a rapid integration^72^. Faster HC growth during that period might thus reflect a beneficial faster sensorimotor and cerebellar growth, since early maturation of these brain structures appears necessary for normal early motor development. Of course, the proposed frameworks for the explanation of our results are speculative, since measuring HC does not allow for quantification of specific brain volumes nor network integration measures. Our results nonetheless contribute to the growing body of evidence on the mechanisms that might underlie early postnatal brain development and their influence later in early childhood.

Interestingly, our results suggest predictive value of general HC growth during the first year of life is sex-specific for developmental outcomes in early childhood. Indeed, overall HC growth from 0 to 12 months predicted temperamental surgency and effortful control, as well as gross motor skills at 24 months in boys, but did not predict any developmental outcomes in girls. This difference may stem from the sexual dimorphism observed in brain structure and brain growth during infancy. Research has shown that there is a disparity in brain size between male and female infants that increases with age, with males growing faster than females^66^, and that males have larger total brain volumes than females in infancy^67,68^. Hence, this more rapid brain growth in boys may potentiate its effect on brain functions and behavior and the likelihood of finding significant results.

Research on the sex disparity in neurodevelopmental disorders also sheds light on an alternative explanation for the sex differences in our results. Indeed, a higher prevalence in males has been repeatedly reported in a number of neurodevelopmental disorders involving attentional difficulties and withdrawal^69,70^, and emerging evidence suggests a “female protective model” as an explanation for the lower prevalence in females^71,72^. This model suggests that a complex mix of genetic factors specific to females might influence neurobehavioral development and act as protective factor even in the presence of adverse circumstances, such as high familial risk for neurodevelopmental disorders. Hence, it could be that brain growth during the first year of life is a less salient biomarker in girls than in boys due to these genetic factors.

HC growth did not predict cognitive skills at 24 months of age. At that young age, specifically assessing cognitive skills with the Bayley-III proves difficult, due to the wide variability of behaviors exhibited by toddlers. Hence, scales or tests targeting more specific cognitive skills in young children, such as attention, memory or specific learning mechanisms, might yield different results. Our study also did not provide support for HC growth during the first year of life as a predictor of temperamental negative affect and fine motor skills at 24 month. The brain correlates underlying these developmental constructs mostly develop in late childhood and adolescence^73–76^, which probably makes HC growth during the neonatal period a less salient biomarker for fine motor skills and negative affect. Finally, it is important to mention that, as opposed to postnatal HC growth, HC at birth does not appear to be a significant predictor of early child functioning in boys or in girls. This is not surprising, since it has been demonstrated that it is not the brain size at a fixed time point that is informative, but rather the evolution between two or more time points that gives a better view of ongoing processes, even more so in infancy where changes are rapid^62,66^. Noteworthy, our sample is representative of a healthy population, thus HC at birth in clinical samples may be relevant to later outcomes^15,77^.

An important strength of this study was our ability to examine associations between postnatal HC growth and early childhood functioning using data from a prospective longitudinal study. Our subsample had a higher SES (i.e. family income and education level) than the general population, which limits the generalizability of our findings. The generalizability to rural populations might also be limited, since the 3D cohort was recruited in large metropolitan areas. Finally, mothers from the 3D cohort study and both our subsamples were older, were more frequently married and more frequently born outside of Canada compared to Canadian births overall^37^.

While an important strength of our study was the assessment of HC growth specifically during the first year of life, HC measurements were taken at only three time points during the first year (birth, 3 months and 12 months). Thus, we may not be able to draw a sufficiently detailed portrait of the accelerated head and brain growth happening in the first year of life. It is also important to stress that HC measurement can only estimate intracranial and overall brain volume. It will thus be important for future studies to assess HC during the postnatal period concurrently with measurement of brain volume to better understand how the different cortical and subcortical brain structures influence HC growth and may affect child development later in life.

## Conclusion

In summary, our study is the first to demonstrate an association between postnatal HC growth and specific behavioral traits during early childhood in a healthy population. We have shown that HC growth during the first year of life predicts specific aspects of temperament, namely effortful control and surgency/extraversion, and gross motor skills in healthy boys at 24 months of age, strengthening the existing evidence that HC is a useful tool to assess during infancy. Further research is warranted to reveal the mechanisms underlying the observed relationships and to support the clinical importance of the findings. The results nonetheless stress the importance of further investigating HC growth during the neonatal period as a biomarker of later child development and strengthen its relevance in clinical populations.
